# Lifestyle risk factors and residual life expectancy at age 40: a German cohort study

**DOI:** 10.1186/1741-7015-12-59

**Published:** 2014-04-07

**Authors:** Kuanrong Li, Anika Hüsing, Rudolf Kaaks

**Affiliations:** 1 Division of Cancer Epidemiology, German Cancer Research Centre, Heidelberg, Germany

**Keywords:** Lifestyle risk factors, Residual life expectancy, Cohort study

## Abstract

**Background:**

Cigarette smoking, adiposity, unhealthy diet, heavy alcohol drinking and physical inactivity together are associated with about half of premature deaths in Western populations. The aim of this study was to estimate their individual and combined impacts on residual life expectancy (RLE).

**Methods:**

Lifestyle and mortality data from the EPIC-Heidelberg cohort, comprising 22,469 German adults ≥40 years and free of diabetes, cardiovascular disease and cancer at recruitment (1994–1998), were analyzed with multivariable Gompertz proportional hazards models to predict lifetime survival probabilities given specific baseline status of lifestyle risk factors. The life table method was then used to estimate the RLEs.

**Results:**

For 40-year-old adults, the most significant loss of RLE was associated with smoking (9.4 [95% confidence interval: 8.3, 10.6] years for male and 7.3 [6.0, 8.9] years for female heavy smokers [>10 cigarettes/day]; 5.3 [3.6, 7.1] years for men and 5.0 [3.2, 6.6] years for women smoking ≤10 cigarettes/day). Other lifestyle risk factors associated with major losses of RLE were low body mass index (BMI <22.5 kg/m^2^, 3.5 [1.8, 5.1] years for men; 2.1 [0.5, 3.6] years for women), obesity (BMI ≥30, 3.1 [1.9, 4.4] years for men; 3.2 [1.8, 5.1] years for women), heavy alcohol drinking (>4 drinks/day, 3.1 [1.9, 4.0] years for men), and high processed/red meat consumption (≥120 g/day, 2.4 [1.0, 3.9] years for women). The obesity-associated loss of RLE was stronger in male never smokers, while the loss of RLE associated with low BMI was stronger in current smokers. The loss of RLE associated with low leisure time physical activity was moderate for women (1.1 [0.05, 2.1] years) and negligible for men (0.4 [−0.3, 1.2] years). The combined loss of RLE for heavy smoking, obesity, heavy alcohol drinking and high processed/red meat consumption, versus never smoking, optimal BMI (22.5 to 24.9), no/light alcohol drinking and low processed/red meat consumption, was 17.0 years for men and 13.9 years for women.

**Conclusions:**

Promoting healthy lifestyles, particularly no cigarette smoking and maintaining healthy body weight, should be the core component of public health approaches to reducing premature deaths in Germany and similar affluent societies.

## Background

In many industrialized countries, basic lifestyle risk factors, such as smoking, adiposity, unhealthy diet, heavy alcohol drinking and lack of physical activity, have been causing a large part of the premature deaths among adults. According to published data, the population attributable fraction (PAF) for smoking alone is about 28% [1,2], for excess body weight about 14% [2], for unhealthy diet 9% to 16% [2,3], for lack of physical activity 7% to 16% [2,4,5], and for heavy alcohol drinking 3% to 18% [2,6]. The estimated PAF of premature mortality for these risk factors combined varies from 42% to 60% in Western populations [2,7-9].

In addition to the PAF estimates, losses of residual life expectancy (RLE) associated with lifestyle risk factors seem to be more meaningful given the fact that death is an inevitable outcome and premature death is difficult to define. The losses of RLE associated with some of the above-mentioned lifestyle factors, most often cigarette smoking and obesity, have been reported by separate studies [10-22]. However, most of these studies did not fully control for mutual confounding effects of lifestyle behaviors. More importantly, no study has so far examined the association between losses of RLE and multiple lifestyle risk factors combined.

In the present study, our aim was to estimate the losses of RLE associated with, individually and jointly, five important lifestyle risk factors, namely cigarette smoking, alcohol drinking, high/low body weight, diet and lack of physical activity, using multivariable Gompertz proportional hazards (PH) regression and the life table method.

## Methods

### Study population

The present study was based on an ongoing cohort study in Heidelberg, Germany – a part of the European Prospective Investigation into Cancer and Nutrition (EPIC) [23]. A total of 11,928 men and 13,612 women, mostly 40-year-old or older, were recruited into the Heidelberg cohort from 1994 to 1998. A detailed description of the recruitment procedures has been published elsewhere [24]. The study protocol was approved by the local ethics committee and all cohort participants provided informed consent. In this study, we excluded participants who had pre-existing diabetes, cardiovascular disease or cancer at baseline (n = 3,064). We further excluded 7 participants without complete data, and eventually we had 22,469 participants (10,235 men and 12,234 women) for statistical analyses.

### Assessment of lifestyle risk factors

Detailed information on past and current smoking was collected at baseline with a computer-guided interview [25]. According to this information, we categorized smoking status at baseline into five groups: never smokers (reference), long-term quitters (duration of cessation >10 years), short-term quitters (duration of cessation ≤10 years), current light smokers (≤10 cigarettes/day), and current heavy smokers (>10 cigarettes/day).

Data on daily consumption of alcoholic beverages at age 20, 30, 40, 50 and over the 12 months before recruitment were collected by means of a self-administered questionnaire. Alcohol intake was derived using the German Food Code and Nutrient Data Base BLS II.3 (BgVV, Berlin, Germany). Average lifetime alcohol intake was estimated as a weighted average of intakes at different ages, with weights equal to the times of exposure to alcohol at different ages. We converted the average lifetime alcohol intake into standard drinks by assuming that one standard drink contains 12 grams of alcohol. For men, we categorized their daily alcohol intake into three groups: ≤2 (reference), 2.1 to 4, and >4 drinks. For women, the three groups were ≤0.5 (reference), 0.6 to 1, and >1 drink.

Anthropometric data were collected during a baseline physical examination. We used the body mass index (BMI, body weight in kilograms divided by the square of height in meters) to categorize baseline body weight status into four groups: optimal BMI (22.5 to 24.9 kg/m^2^, reference), low BMI (<22.5), overweight (BMI 25 to 29.9), and obese (BMI ≥30). The choice of BMI 22.5 to 24.9 as the optimal group was made based on the finding from large cohort data showing the lowest mortality rate for this group [26].

Occupational, household and recreational activity was assessed at baseline using a short questionnaire [27]. The intensity of physical activity was measured with metabolic equivalent (MET) values. A MET is defined as the ratio of work metabolic rate to a standard metabolic rate of 1.0 (4.184 kJ) kg^−1^.h^−1^. For each reported activity, a MET value was assigned according to the Compendium of Physical Activity [28]. A large meta-analysis of cohort studies has shown a positive association between leisure time physical activity and life expectancy [19]. Therefore, we were particularly interested in leisure time physical activity, which in the present study was a combination of walking, cycling and other sports. We chose the median of leisure time activity in MET-hours/week to categorize participants into the low group (<36 MET-hours/week) and the high group (≥36 MET-hours/week, reference).

Data on usual consumption levels of 148 food items over the last 12 months before recruitment were collected using a food frequency questionnaire (FFQ) validated by 24-hr dietary recalls. The validity and reproducibility of the FFQ were acceptable, with the correlation coefficients varying from 0.21 for fish to 0.90 for alcoholic beverages [29]. In the present study, we focused on potentially important food groups, namely, processed/red meat, vegetables/fruits, cereals, fish, and dairy products, which, according to previous studies [30-34], might have an association with mortality rates. We dichotomized the consumption of these food groups, except processed/red meat, as high and low by using their medians as the cut-off points. For processed/red meat, we chose 120 g/day as the cut-off point, as it has been suggested that a higher consumption than this amount may significantly increase the mortality rate [30]. Besides these selected food items, a more exploratory analysis covering a total of 16 finer food groups and other dietary factors showed no additional associations with the risk of premature death [see Additional file 1: Table S1].

### Ascertainment of deaths

Information on vital status was collected through the official death registry system and reports from next of kin. All reported deaths were verified by obtaining official death certificates. In the present study, we analyzed the mortality data until December 31, 2009, by which time the vital status of all cohort participants had been completely ascertained.

### Statistical analysis

Mortality data were analyzed with two, sex-specific multivariable Gompertz PH models that included smoking status, body weight status, alcohol drinking, consumption of the selected food groups (processed/red meat, vegetables/fruits, cereals, fish and dairy products) and leisure time physical activity. Education and self-reported pre-existing hypertension and hyperlipidemia were additionally included as confounders.

For each of the lifestyle risk factors, we detected no departure from the proportionality assumption by checking the parallelity of the log-log survival plots. Age was modeled as the time scale. As our aim was to estimate the RLE at age 40, we left-truncated the data at this age. Unlike the widely used Cox PH model that leaves the baseline hazard function unspecified, Gompertz PH model is parametric and assumes that the baseline hazard is an exponential function of age.

We predicted lifetime survival probabilities with the multivariable Gompertz PH models given specific baseline status of the lifestyle risk factors. The maximum age attained by cohort participants at the end of the follow-up was 82 years; however, we extrapolated the prediction up to the age of 110 years, which was assumed to be the maximum age that any of our cohort participants could theoretically attain. As the multivariable Gompertz PH models showed no statistically significant association with mortality rate for cereals, fish and dairy products in both sexes, we fixed these factors at the assumedly healthy level (high consumption of cereals and fish but low consumption of dairy products) when we calculated the lifetime survival probabilities. In addition, we fixed the educational degree at the intermediate level (‘secondary/professional’) and pre-existing hypertension and hyperlipidemia at “no”. Assigning a fixed value to these factors, however, did not change the estimated losses of RLE in relation to the other lifestyle risk factors.

We applied the life table method to convert the predicted lifetime survival probabilities into RLEs. In brief, we used the predicted lifetime survival probability on a hypothetical cohort of 100,000 40-year-old subjects to calculate the expected number of deaths (*d*_*t*_) that would occur within each age interval [*t*, *t*+1). The number of person-years of survival within [*t*, *t*+1), denoted as *L*_*t*_, given the number of subjects who remained alive at age *t*, denoted as *l*_*t*_, was estimated as follows:

$$L_{t}=l_{t}-0.5\times d_{t}$$

The total person-years of survival remaining at age 40 (*T*_40_) was calculated by summing up *L*_*t*_ from the last age interval, [100, 110), backward to the age interval [40, 41). The RLE at age 40 (*RLE*_40_) was then calculated as:

$$\mathrm{RL}E_{40}=T_{40}/100,000$$

The loss of RLE associated with a lifestyle risk factor was calculated as the difference between the RLEs given the absence and the presence of this factor, respectively, while keeping other factors identical. We estimated 95% confidence intervals for the losses of RLE using the bootstrap method.

As low body weight is likely to be a result of undiagnosed diseases, we performed a sensitivity analysis to examine the possible reverse causality by excluding deaths that occurred within the first two years of the follow-up period. Since previous research shows a stronger obesity-mortality association in never smokers than in current smokers [35,36], we also examined the effect of body weight on RLE across the smoking categories. To ensure sufficient cases in each category, we combined all the quitters as former smokers and light and heavy smokers as current smokers. We did not consider possible effect modification for other pairwise risk factor combinations because of lack of both biological and statistical evidence (the minimum *P*-value for interaction was 0.02, which was not regarded as significant evidence from multiple comparisons).

For the male and female sub-cohorts, we predicted their lifetime survival probabilities using a Gompertz PH model that did not include any covariates, and then compared the predicted survival probabilities with the observed ones, which were estimated with the life table method. We also compared the trajectories of the extrapolated survival curves of our cohort with those of the general German population. The latter were derived from the German life table [37].

All statistical analyses were performed with SAS (version 9.2, SAS Institute, Cary, NC, USA) and the package ‘eha’ in R (version 3.0.1, R Foundation for Statistical Computing, Vienna, Austria). Two-sided *P*-values <0.05 were considered statistically significant.

## Results

After an average follow-up time of 11 years (range 0.1 to 19), we documented 1,599 deaths, 1,040 among men and 559 among women. The distribution of lifestyle risk factors and other characteristics as assessed at baseline is given in Table 1.

**Table 1:** **Baseline distribution of lifestyle risk factors, the EPIC-Heidelberg cohort**^**a**^

	**Men (n = 10,235)**	**Women (n = 12,234)**
Age, mean (range)	51.9 (40, 65.8)	49.3 (40, 66.0)
Smoking category (%)		
Never smokers	3,156 (30.8)	5,808 (47.5)
Long-term quitters (>10 years)	3,414 (33.4)	2,384 (19.5)
Short-term quitters (≤10 years)	1,206 (11.8)	1,194 (9.8)
Light smokers (≤10 cigarettes/day)	638 (6.2)	1,228 (10.0)
Heavy smokers (>10 cigarettes/day)	1,821 (17.8)	1,620 (13.2)
Body weight status (%)		
Optimal BMI (22.5 to 24.9 kg/m^2^)	2,379 (23.2)	3,168 (25.9)
Low BMI (<22.5 kg/m^2^)	913 (8.9)	3,728 (30.5)
Overweight (BMI 25 to 29.9 kg/m^2^)	5,262 (51.5)	3,595 (29.4)
Obesity (BMI ≥30 kg/m^2^)	1,681 (16.4)	1,743 (14.2)
Alcoholic drinks/day (%)		
≤2 (men)/ ≤0.5 (women)	6,060 (59.2)	7,278 (59.5)
2.1 to 4 (men)/ 0.6 to 1 (women)	2,700 (26.4)	2,649 (21.7)
>4 (men)/ >1 (women)	1,475 (14.4)	2,307 (18.8)
Leisure time physical activity (%)		
High (≥36 METS-hours/week)	4,853 (47.4)	6,043 (49.4)
Low (<36 METS-hours/week)	5,382 (52.6)	6,191 (50.6)
Processed/red meat consumption (%)		
Low (<120 g/day)	6,912 (67.5)	11,118 (90.9)
High (≥120 g/day)	3,323 (32.5)	1,116 (9.1)
Vegetable/fruit consumption (%)		
High (≥200 g/day)	5,200 (50.8)	7,751 (63.4)
Low (<200 g/day)	5,035 (49.2)	4,483 (36.6)
Cereal consumption (%)		
High (≥200 g/day)	5,185 (50.7)	3,931 (32.1)
Low (<200 g/day)	5,050 (49.3)	8,303 (67.9)
Fish consumption (%)		
High (≥15 g/day)	6,363 (62.2)	6,066 (49.6)
Low (<15 g/day)	3,872 (37.8)	6,168 (50.4)
Dairy product consumption (%)		
Low (<200 g/day)	5,797 (56.6)	5,961 (48.7)
High (≥200 g/day)	4,438 (43.4)	6,273 (51.3)
Education level (%)		
No/primary school	3,067 (30.0)	3,176 (26.0)
Secondary/professional	3,288 (32.1)	5,965 (48.7)
University	3,880 (37.9)	3,093 (25.3)
Self-reported hypertension (%)		
No	6,886 (67.3)	9,435 (77.1)
Yes	3,349 (32.7)	2,799 (22.9)
Self-reported hyperlipidemia (%)		
No	5,989 (58.5)	8,769 (71.7)
Yes	4,246 (41.5)	3,465 (28.3)

^a^Participants with pre-existing diabetes, cardiovascular disease or cancer were excluded. BMI, body mass index; EPIC, European Prospective Investigation into Cancer and Nutrition; METS, metabolic equivalents.

Table 2 shows the associations between lifestyle risk factors and all-cause mortality from univariate and multivariable Gompertz PH models. For men, overweight and low cereal consumption showed statistically significant associations with all-cause mortality in the univariate model but not in the multivariable model. For women, this dilution was observed for high alcohol intake (>1 drink/day), low leisure time physical activity, low vegetable/fruit consumption and low fish consumption. In multivariable models, a statistically significant association was observed for heavy and light smoking, low BMI, obesity and high processed/red meat consumption for both men and women. Former cigarette smoking, heavy alcohol drinking (>4 drinks/day), and low vegetable/fruit consumption were additional significant risk factors for men. Low leisure time physical activity was associated with a non-significant increase in all-cause mortality for both sexes.

**Table 2:** **HR (95% CI) of lifestyle risk factors for all-cause mortality, the EPIC-Heidelberg cohort**^**a**^

	**Men (n = 10,235)**			**Women (n = 12,234)**		
	**Deaths**	**Univariate**	**Multivariable**^**b**^	**Deaths**	**Univariate**	**Multivariable**^**b**^
Smoking category						
Never smokers	209	1.00	1.00	273	1.00	1.00
Long-term quitters	313	1.29 (1.09, 1.54)	1.21 (1.01, 1.44)	84	1.01 (0.79, 1.29)	1.03 (0.80, 1.32)
Short-term quitters	118	2.13 (1.70, 2.68)	1.87 (1.49, 2.35)	33	1.10 (0.77, 1.58)	1.14 (0.79, 1.64)
Light smokers	71	2.26 (1.73, 2.96)	2.02 (1.54, 2.65)	66	1.93 (1.47, 2.53)	2.00 (1.52, 2.63)
Heavy smokers	329	4.20 (3.53, 5.00)	3.49 (2.90, 4.18)	103	2.82 (2.23, 3.55)	2.75 (2.16, 3.48)
Body weight status						
Optimal BMI	164	1.00	1.00	114	1.00	1.00
Low BMI	90	1.78 (1.37, 2.30)	1.61 (1.23, 2.07)	127	1.38 (1.07, 1.78)	1.34 (1.04, 1.73)
Overweight	535	1.28 (1.08, 1.53)	1.16 (0.97, 1.38)	185	1.10 (0.87, 1.39)	1.08 (0.85, 1.37)
Obesity	251	1.83 (1.50, 2.22)	1.51 (1.23, 1.86)	133	1.56 (1.21, 2.00)	1.55 (1.19, 2.02)
Alcoholic drinks/day						
≤2 (men)/ ≤0.5 (women)	494	1.00	1.00	343	1.00	1.00
2.1 to 4 (men)/ 0.6 to 1 (women)	299	1.36 (1.18, 1.57)	1.24 (1.07, 1.44)	101	0.87 (0.70, 1.09)	0.86 (0.69, 1.08)
>4 (men)/ >1 (women)	247	2.07 (1.78, 2.42)	1.50 (1.28, 1.75)	115	1.24 (1.00, 1.53)	1.15 (0.92, 1.44)
Leisure time physical activity						
Low versus high	555/485	1.11 (0.98, 1.26)	1.05 (0.93, 1.19)	310/249	1.24 (1.05, 1.46)	1.16 (0.98, 1.37)
Processed/red meat consumption						
High versus low	393/647	1.43 (1.26, 1.62)	1.21 (1.06, 1.38)	72/487	1.54 (1.20, 1.97)	1.40 (1.08, 1.80)
Vegetable/fruit consumption						
Low versus high	578/462	1.32 (1.16, 1.49)	1.19 (1.05, 1.35)	222/337	1.19 (1.01, 1.41)	1.11 (0.93, 1.32)
Cereal consumption						
Low versus high	596/444	1.19 (1.06, 1.35)	1.08 (0.96, 1.23)	407/152	1.10 (0.91, 1.33)	1.04 (0.86, 1.26)
Fish consumption						
Low versus high	427/613	1.11 (0.98, 1.26)	1.04 (0.91, 1.18)	296/263	1.20 (1.02, 1.42)	1.17 (0.99, 1.39)
Dairy product consumption						
High versus low	407/633	0.92 (0.81, 1.05)	1.07 (0.95, 1.22)	277/282	0.95 (0.81, 1.12)	1.02 (0.86, 1.21)

^a^Participants with pre-existing diabetes, cardiovascular disease or cancer were excluded. ^b^The lifestyle risk factors were mutually adjusted for. Education and self-reported hypertension and hyperlipidemia were also adjusted for as confounding factors. BMI, body mass index; CI, confidence interval; EPIC, European Prospective Investigation into Cancer and Nutrition; HR, hazard ratio.

The RLEs associated with all possible combinations of lifestyle risk factors are presented in Additional file 2: Table S2 (men) and Additional file 3: Table S3 (women). For 40-year-old men living with the healthiest combination (never smoking, BMI 22.5 to 24.9, no/light alcohol drinking [≤2 drinks/day], high leisure time physical activity, low processed/red meat consumption and high vegetable/fruit consumption), the RLE was estimated to be 47.5 years. For the unhealthiest combination (heavy smoking, obesity, heavy alcohol drinking, low leisure time physical activity, high processed/red meat consumption and low vegetable/fruit consumption), the RLE was estimated to decrease by 18.5 years, to 29.0 years. For 40-year-old women, the RLEs for the two extreme combinations were estimated to be 48.7 years and 33.0 years.

The losses of RLE associated with individual lifestyle risk factors are summarized in Table 3. Because of the PH property of the Gompertz models, the estimated loss of RLE associated with any given lifestyle risk factor alone remains nearly constant. The slight variations (± 0.1) in the estimates can be explained by the fact that all survival curves started at value 1 for age 40 and converged at 0 for age 110. When multiple lifestyle risk factors are present, the combined loss of RLE can be calculated by addition. For example, for 40-year-old adults, the combined loss of RLE associated with heavy smoking, obesity, heavy alcohol drinking and high processed/red meat consumption, versus never smoking, optimal BMI, no/light alcohol drinking and low processed/red meat consumption, was 17.0 (9.4 + 3.1 + 3.1 + 1.4) years for men and 13.9 (7.3 + 3.2 + 1.0 +2.4) years for women.

**Table 3:** **Estimated loss of RLE (95% CI) associated with individual lifestyle risk factors for 40-year-old men and women, the EPIC-Heidelberg cohort**^**a**^

	**Men (n = 10,235)**	**Women (n = 12,234)**
Smoking category		
Never smokers	0	0
Long-term quitters	1.4 (0.3, 2.4)	0.2 (−1.4, 1.9)^b^
Short-term quitters	4.8 (3.3, 6.3)	0.9 (−1.2, 3.2)^b^
Light smokers	5.3 (3.6, 7.1)	5.0 (3.2, 6.6)
Heavy smokers	9.4 (8.3,10.6)	7.3 (6.0, 8.9)
Body weight status		
Optimal BMI	0	0
Low BMI	3.5 (1.8, 5.1)	2.1 (0.5, 3.6)
Overweight	1.1 (0.1, 2.3)	0.6 (−0.8, 2.3)^b^
Obesity	3.1 (1.9, 4.4)	3.2 (1.8, 5.1)
Alcoholic drinks/day		
≤2 (men)/ ≤0.5 (women)	0	0
2.1 to 4 (men)/ 0.6 to 1 (women)	1.7 (0.8, 2.7)	−1.0 (−2.6, 0.3)^b^
>4 (men)/ >1 (women)	3.1 (1.9, 4.0)	1.0 (−0.3, 2.4)^b^
Leisure time physical activity		
High	0	0
Low	0.4 (−0.3, 1.2)^b^	1.1 (0.05, 2.1)
Processed/red meat consumption		
Low	0	0
High	1.4 (0.6, 2.2)	2.4 (1.0, 3.9)
Vegetable/fruit consumption		
High	0	0
Low	1.3 (0.4, 2.1)	0.8 (−0.2, 1.9)^b^

^a^Participants with pre-existing diabetes, cardiovascular disease or cancer were excluded. ^b^Minus sign denotes a gain in RLE. BMI, body mass index; CI, confidence interval; EPIC, European Prospective Investigation into Cancer and Nutrition; RLE, residual life expectancy.

Results from sensitivity analyses are shown in Table 4. The loss of RLE associated with low BMI became slightly lower after deaths that occurred in the first two years of the follow-up period were excluded. After stratification by smoking status, the loss of RLE associated with obesity became stronger in male never smokers (3.1 versus 5.2 years) but remained similar in female never smokers (3.2 versus 3.0 years), while the loss of RLE associated low BMI became stronger in both male and female current smokers (3.5 versus 4.3 and 2.1 versus 4.8 years, respectively).

**Table 4:** **HR (95% CI) and estimated loss of RLE (95% CI) for body weight status, after exclusion of early deaths, and after stratification by smoking status, the EPIC-Heidelberg cohort**^**a**^

	**Men**			**Women**	
	**Multivariable HR (95% CI)**	**Loss of RLE (95% CI)**		**Multivariable HR (95% CI)**	**Loss of RLE (95% CI)**
Early deaths^b^ excluded					
Body weight status					
Optimal BMI	1.00	0		1.00	0
Low BMI	1.51 (1.15, 1.98)	3.0 (1.0, 4.6)		1.30 (1.00, 1.69)	1.8 (0.3, 3.1)
Overweight	1.14 (0.95, 1.37)	1.0 (0, 2.0)		1.02 (0.79, 1.30)	0.1 (−1.1, 1.7)^c^
Obesity	1.49 (1.21, 1.84)	2.9 (1.8, 4.1)		1.48 (1.13, 1.93)	2.6 (1.2, 4.3)
Stratified by smoking status					
Never smokers					
Optimal BMI	1.00	0		1.00	0
Low BMI	1.50 (0.80, 2.82)	2.9 (−2.5, 6.4)^c^		1.05 (0.71, 1.55)	0.4 (−2.4, 2.8)^c^
Overweight	1.15 (0.77, 1.72)	1.0 (−1.5, 3.7)^c^		0.97 (0.69, 1.36)	−0.2 (−2.0, 2.1)^c^
Obesity	2.05 (1.31, 3.21)	5.2 (2.1, 8.6)		1.53 (1.06, 2.19)	3.0 (0.9, 5.2)
Former smokers					
Optimal BMI	100	0		1.00	0
Low BMI	1.32 (0.77, 2.29)	2.0 (−1.6, 5.4)^c^		1.14 (0.63, 2.08)	0.9 (−3.1, 4.4)^c^
Overweight	1.36 (1.01, 1.83)	2.2 (0.6, 4.4)		1.41 (0.86, 2.32)	2.3 (−0.6, 5.2)^c^
Obesity	1.55 (1.10, 2.18)	3.1 (1.2, 5.4)		1.37 (0.75, 2.48)	2.1 (−1.1, 5.6)^c^
Current smokers					
Optimal BMI	1.00	0		1.00	0
Low BMI	1.70 (1.21, 2.40)	4.3 (1.7, 6.6)		1.83 (1.19, 2.82)	4.8 (2.1, 8.0)
Overweight	1.00 (0.77, 1.31)	0 (−1.9, 2.0)^c^		1.04 (0.66, 1.63)	0.3 (−2.6, 3.4)^c^
Obesity	1.39 (1.00,1.92)	2.7 (0.6, 5.2)		1.68 (1.01, 2.80)	4.2 (0.6, 7.6)

^a^Participants with pre-existing diabetes, cardiovascular disease or cancer were excluded. ^b^Deaths occurred in the first two years of the follow-up period (n_men_ = 48; n_women_ = 33). ^c^Minus sign denotes a gain in RLE. BMI, body mass index; CI, confidence interval; EPIC, European Prospective Investigation into Cancer and Nutrition; HR, hazard ratio; RLE, residual life expectancy.

**Figure 1:**
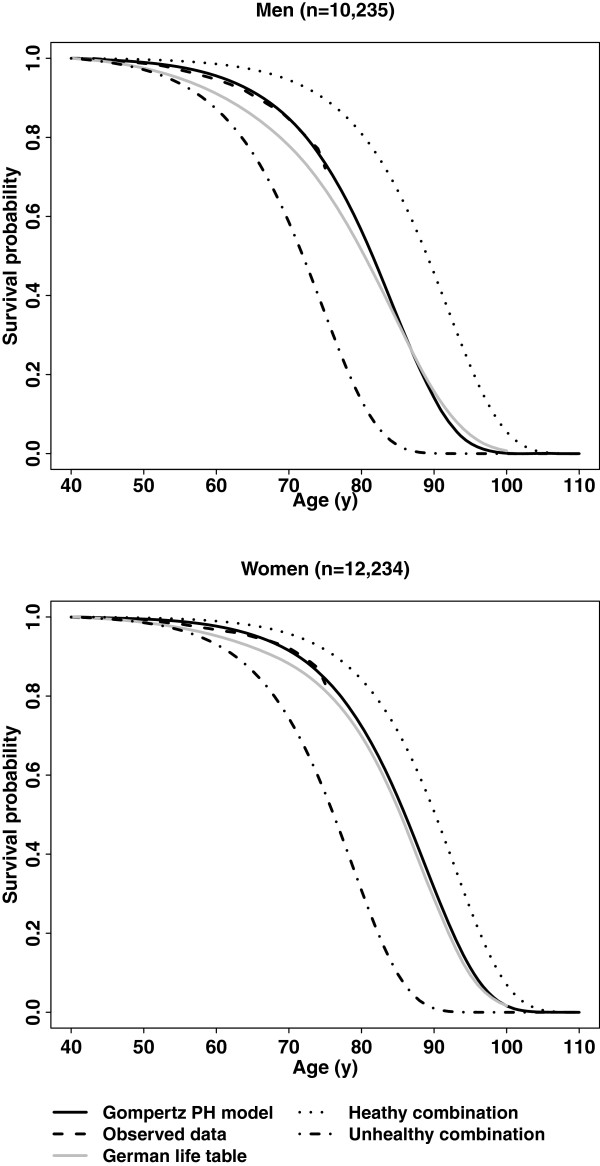
**Overall survival curves (Gompertz PH models, observed data, and the German life table) and two survival curves for the healthy and the unhealthy combination of lifestyle risk factors, respectively, the EPIC-Heidelberg cohort**^**a**^**.**^a^Participants with pre-existing diabetes, cardiovascular disease or cancer were excluded. The healthy combination: never smoking, optimal BMI (22.5 to 24.9), no/light alcohol drinking and low processed/red meat consumption; the unhealthy combination: heavy smoking (>10 cigarettes/day), obesity (BMI ≥30), heavy alcohol drinking (>4 drinks/day for men and >1 drink/day for women) and high processed/red meat consumption (≥120 g/day). Other factors remained identical for these two groups and were fixed at their assumedly healthy level. BMI, body mass index; EPIC, European Prospective Investigation into Cancer and Nutrition; PH, proportional hazards.

As shown in Figure 1, the predicted survival probabilities based on the Gompertz PH models without covariates fit the observed survival probabilities very well within the prospective observation time of the cohort. The extrapolated lifetime survival curves for our cohort and the lifetime survival curves for the general German population also showed similar trajectories, although the survival probabilities for our cohort were overall a bit higher.

## Discussion

This study shows substantial losses of RLE at age 40 in relation to cigarette smoking, unhealthy body weight and high processed/red meat consumption for both sexes and heavy alcohol drinking for men. In contrast, the losses of RLE associated with low leisure time physical activity and low vegetable/fruit consumption were relatively modest and inconsistent between men and women.

This study provides clear evidence that cigarette smoking is the lifestyle behavior most strongly reducing longevity. In this study, heavy smoking alone is expected to reduce the RLE by about nine years for men and seven years for women. Other cohort-based estimates of smoking-related loss of RLE, mostly from Japanese studies, vary from two to ten years [10-13]. None of these latter studies, however, performed a sufficient control for potential confounding effects of other important lifestyle risk factors and non-lifestyle risk factors, such as baseline health status and socio-economic status.

Our study further suggests the importance of early quitting for men by showing a loss of 1.4 years in RLE for long-term quitters in contrast to 4.8 years for short-term quitters. The difference in the loss of RLE between female long-term and short-term quitters is slight (0.2 versus 0.9 years). This might be explained by the fact that female smokers on average smoke fewer cigarettes per day, and, thus, the cumulative impact of smoking on their RLE was rather modest, making the benefit of early quitting much less evident.

According to previous studies, the loss of RLE associated with excess body weight varied from less than one year to about seven years, and as high as thirteen years for severe cases (BMI >45) [14-18], without considering the confounding effects of other key lifestyle factors. Taking confounding effects of smoking and other relevant lifestyle risk factors into account, we found that obesity alone was associated with a loss of RLE of about three years for both men and women. Another important difference between our study and previous studies is that we adjusted for pre-existing obesity-related conditions (hypertension and hyperlipidemia) and our subjects were also free of pre-existing diabetes, cardiovascular disease and cancer.

In our study, overweight is related to a loss of RLE of 1.1 (95% CI: 0.1 to 2.3) years for men. This finding does not support the conclusion from a recent pooled analysis of cohort data, in which a 6.0% lower risk of premature death was observed for overweight people compared to people with a BMI of 18.5 to 24.9 [38]. However, many individual studies have shown a J-shaped relationship between BMI and all-cause mortality. The reference category in the pooled analysis (BMI 18.5 to 24.9) may have included some subjects at increased risk of death because of low body weight, and, therefore, it is reasonable to assume that the reported protective effect of overweight against all-cause mortality might disappear or even reverse if the optimal BMI (22.5 to 24.9) was used as the reference. The overweight-related loss of RLE is only modest in itself, but the overall loss of RLE at population level can be considerable given the high prevalence of overweight among our male participants (51.5%).

The present study also suggests that low body weight may cause a similar loss of RLE for men as obesity does, despite the fact that the former has been largely overlooked in societies where obesity is more prevalent. It should be noted that in our low BMI group, the average BMI was 21.2 for men and 20.8 for women, and only a few participants (22 men and 17 women) would be categorized as low body weight if we used the WHO criterion (BMI <18.5). In addition, our study population was free of major chronic diseases including cancer, and after a further exclusion of deaths that occurred in the first two years of the follow-up period, low BMI was still associated with a similar loss of RLE, which is, therefore, unlikely to be a result of reverse causality.

By showing a stronger loss of RLE associated with low BMI in current smokers than in never smokers and a stronger loss of RLE associated with obesity in male never smokers, our study confirms that smoking is a likely effect modifier in the association between adiposity and mortality, which might be explained by the association between smoking and leanness and smoking itself as a risk factor for increased mortality.

Our study confirms the harmful effect of heavy alcohol drinking by showing a loss of 3.1 years in RLE among men who were heavy drinkers. However, a large-scale meta-analysis of prospective cohort data indicated that intake of one to two drinks/day for women and two to four drinks/day for men may reduce the risk of all-cause mortality by about 18% [39]. One previous study also reported a 1.9 year longer life expectancy for men with an average daily alcohol intake of 29 grams, as compared to non-drinkers [22]. Our study, however, shows a loss of 1.7 years in RLE for men drinking roughly the same amount of alcohol. This inconsistency might be due to different ways of measuring alcohol intake. In the present study, lifetime alcohol intake was used, while in other studies alcohol intake measured at baseline was used. For women, we did see an increased RLE by one year in relation to light alcohol consumption (0.6 to 1 drink/day), which was, however, not statistically significant. We did not consider heavier drinkers (for example, > 4 drinks/day) in our female sub-cohort as they were really few.

According to a pooled analysis of cohort data, no leisure time physical activity is associated with a loss of RLE by more than 4 years in men and women when compared with leisure time physical activity of more than 22.5 MET-hours/week [19]. In the present study, we found a much less evident loss in RLE by low leisure time physical activity (<36 MET-hours/week, 0.4 year for men and 1.1 year for women). Our study population was overall physically active, and there were only a few subjects with very low leisure time physical activity. Possibly, we failed to observe a more impressive loss in RLE due to the choice of the higher reference. It should be noted that current methods for physical activity assessment, including the method used in the EPIC cohort, are subject to substantial measurement errors, and the definition of leisure time physical activity also may not be the same across studies, rendering inter-study comparisons difficult.

Among food groups that may potentially influence the RLE, we only found a solid, negative association between RLE and processed/red meat consumption for both men and women. This finding supports the result of a recent meta-analysis that shows a 29% higher mortality rate (95% CI: 1.20 to 1.38) for processed/red meat consumption of 120 g/day versus 20 g/day [30]. Low fruit/vegetable consumption has also been associated with an increased mortality rate in a study based on the entire EPIC cohort [31], but we only confirmed this finding among men by showing a loss of RLE by 1.3 (95% CI: 0.4 to 2.1) years associated with the low consumption. Our study did not replicate the previously reported associations of high consumption of cereals, fish and low consumption of dairy products with all-cause mortality. However, the absence of independent associations of diet with mortality could be due to inaccuracies in the dietary intake assessments, and we do not rule out that the RLE could be further extended by adhering to the current diet guidelines.

According to the present study, the combined loss of RLE for heavy smoking, obesity, heavy alcohol drinking and high processed/red meat consumption, in contrast to never smoking, optimal BMI, no/light alcohol drinking and low processed/red meat consumption, was 17.0 years for men and 13.9 years for women. This is a strong message to advocate for healthy lifestyle behaviors and favorable lifestyle modification. By maintaining an optimally healthy lifestyle, the life expectancy of German men and women would reach about 87.5 and 88.7 years, respectively, which is significantly higher than the current estimates (about 78 years for men and 82 years for women). Our results also suggest that the current gender gap in life expectancy can be largely explained by the higher prevalence of harmful lifestyle behaviors among men.

The validity of our findings depends somewhat on several underlying model assumptions. The first assumption is the proportionality hypothesis, that is, the effects of lifestyle risk factors are supposed to be constant over time. Although we did not see a violation of this assumption, we still could not preclude the possibility of time-varying effects of some risk factors, such as the well-known ‘obesity paradox.’ The second assumption is the Gompertz distribution of the baseline hazard function for all-cause mortality, which describes an exponential increase of mortality rate with age. This distribution has been widely applied to study human mortality and life span [40]. In the present study, the predicted survival probabilities by the Gompertz PH models show a good internal consistency with the observed survival probabilities, suggesting that the Gompertz law works reasonably well with our mortality data. Secondly, because our prospective observation was only up to a maximum age of 82 years for our oldest study participants, we extrapolated the Gompertz survival curves in order to predict the lifetime survival probabilities. The trajectories of the extrapolated survival curves for our cohort and those for the general German population are very close, and, therefore, we believe that our estimates on the losses of RLE associated with basic lifestyle risk factors may be quite generalizable to the German population as a whole. Finally, the validity of our findings also relies on the accuracy of risk factor assessments. However, some lifestyle risk factors, such as habitual diet and physical activity, are known for substantial measurement errors, which may cause an underestimation of their impacts on RLE.

## Conclusions

The present study finds that substantial losses of RLE can be associated with cigarette smoking, high/low body weight, heavy alcohol drinking and high processed/red meat consumption, confirming that promoting healthy lifestyles, particularly no cigarette smoking and maintaining healthy body weight, should be the core component of public health approaches to reducing premature deaths in Germany and similar affluent societies.

## Competing interests

The authors declare that they have no competing interests.

## Authors’ contributions

Study concept and design: KL, RK, and AH. Statistical analysis: KL. Result interpretation: KL, RK, and AH. Drafting of the manuscript: KL. Critical revision of the manuscript for important intellectual content: RK and AH. Obtained funding: RK. All authors read and approved the final manuscript.

## Pre-publication history

The pre-publication history for this paper can be accessed here:

http://www.biomedcentral.com/1741-7015/12/59/prepub

## Supplementary Material

Additional file 1: Table S1 HR (95% CI) of 16 food groups and dietary factors for all-cause mortality, the EPIC-Heidelberg cohort. Click here for file

Additional file 2: Table S2 Estimated RLEs for 40-year-old men, based on the EPIC-Heidelberg male sub-cohort (n = 10,235). Click here for file

Additional file 3: Table S3 Estimated RLEs for 40-year-old women, based on the EPIC-Heidelberg female sub-cohort (n = 12,234). Click here for file
