# Supplementary material for: Lifestyle risk factors and residual life expectancy at age 40: a German cohort study
Source: BMC Med. 2014 Apr 7;12:59. doi: 10.1186/1741-7015-12-59 (PMC4022368; doi:10.1186/1741-7015-12-59)
Supplement: Additional file 1: Table S1 — HR (95% CI) of 16 food groups and dietary factors for all-cause mortality, the EPIC-Heidelberg cohort. [file 1741-7015-12-59-S1.doc]

| **Suppl. Table 1 HR (95% CI) of 16 food groups and dietary factors for all-cause mortality, the EPIC-Heidelberg cohort** ^a^ | | | | | |
| --- | --- | --- | --- | --- | --- |
|  | Men (n=10,235) | |  | Women(n=12,234) | |
|  | Univariate | Multivariable ^b^ |  | Univariate | Multivariable ^b^ |
| Legumes: low vs. high | 0.81 (0.76, 0.87) | 0.92 (0.80, 1.05) |  | 0.97 (0.82, 1.14) | 0.99 (0.84, 1.17) |
| Leafy vegetables: low vs. high | 1.18 (1.11, 1.26) | 1.13 (1.00, 1.27) |  | 0.95 (0.80, 1.12) | 0.93 (0.78, 1.10) |
| Fruit vegetables: low vs. high | 1.24 (1.16, 1.23) | 1.14 (1.00, 1.29) |  | 1.11 (0.94, 1.31) | 1.07 (0.90, 1.27) |
| Root vegetables: low vs. high | 1.11 (1.04, 1.18) | 0.99 (0.87, 1.12) |  | 1.03 (0.87, 1.22) | 0.95 (0.80, 1.12) |
| Fruits: low vs. high | 1.15 (1.01, 1.30) | 0.99 (0.87, 1.12) |  | 1.05 (0.89, 1.23) | 0.95 (0.81, 1.13) |
| Red meat: high vs. low | 1.23 (1.15, 1.33) | 1.00 (0.86, 1.16) |  | 0.98 (0.83, 1.16) | 0.93 (0.78, 1.10) |
| Processed meat: high vs. low | 1.28 (1.20, 1.37) | 1.05 (0.91, 1.21) |  | 1.19 (1.01, 1.14) | 1.12 (0.94, 1.33) |
| Milk: high vs. low | 0.89 (0.83, 0.94) | 0.95 (0.84, 1.08) |  | 1.05 (0.89, 1.24) | 1.06 (0.89, 1.25) |
| Yogurt: high vs. low | 0.83 (0.78, 0.89) | 1.00 (0.88, 1.14) |  | 0.82 (0.69, 0.97) | 0.88 (0.74, 1.04) |
| Cheese: high vs. low | 0.88 (0.82, 0.93) | 0.97 (0.86, 1.10) |  | 0.93 (0.79, 1.10) | 0.97 (0.82, 1.14) |
| Dietary fiber: low vs. high | 1.21 (1.14, 1.29) | 1.07 (0.95, 1.21) |  | 1.12 (0.94, 1.33) | 1.01 (0.85, 1.21) |
| Total fat: high vs. low | 1.13 (1.06, 1.20) | 1.02 (0.90, 1.16) |  | 1.16 (0.98, 1.38) | 1.13 (0.95, 1.34) |
| Saturated fat: high vs. low | 1.10 (1.03, 1.17) | 1.00 (0.89, 1.14) |  | 1.16 (0.98, 1.37) | 1.12 (0.94, 1.32) |
| Monounsaturated fat: low vs. high | 0.89 (0.84, 0.95) | 1.00 (0.88, 1.14) |  | 0.85 (0.71, 1.01) | 0.88 (0.74, 1.05) |
| Polyunsaturated fat: low vs. high | 0.95 (0.89, 1.02) | 1.00 (0.87, 1.14) |  | 0.96 (0.81, 1.13) | 0.95 (0.80, 1.12) |
| Fat ratio: high vs. low | 0.96 (0.90, 1.02) | 0.97 (0.85, 1.09) |  | 1.02 (0.86, 1.20) | 1.00 (0.84, 1.18) |
| ^a^ Participants with pre-existing diabetes, cardiovascular disease or cancer were excluded.  ^b^ Adjusted for all non-dietary risk factors listed in Table 2. | | | | | |
